# Bacterial factors required for biofilm formation in *Staphylococcus epidermidis* are linked to contact activation

**DOI:** 10.3389/fcimb.2026.1802218

**Published:** 2026-04-14

**Authors:** Annika Rademacher, Katharina Ekat, Romy Skusa, Lotte Scharnagl, Frederike Knipp, Christoph Warnke, Julia Marcinek, Selina Tobien, Marcus Frank, Valeria Khaimov, Holger Rohde, Bernd Kreikemeyer, Sonja Oehmcke-Hecht

**Affiliations:** 1 Institute of Medical Microbiology, Virology and Hygiene, Rostock University Medical Center, Rostock, Germany; 2 Medical Biology and Electron Microscopy Centre, Rostock University Medical Center, Rostock, Germany; 3 Department Life, Light and Matter, University of Rostock, Rostock, Germany; 4 Institute for Implant Technology and Biomaterials e.V., Rostock, Germany; 5 Institute of Medical Microbiology, Virology and Hygiene, University Medical Center Hamburg-Eppendorf, Hamburg, Germany

**Keywords:** AtlE, biofilm, coagulation, contact system, PIA, *Staphylococcus epidermidis*

## Abstract

*Staphylococcus epidermidis* is a leading cause of device-associated bloodstream infections, where biofilm formation contributes to persistence in direct contact with host plasma. While extracellular matrix components are central to biofilm development, their functional consequences at the host–pathogen interface remain incompletely understood. Here, we investigated whether bacterial factors required for biofilm formation are associated with activation of the intrinsic coagulation pathway in human plasma. Clinical isolates of *S. epidermidis* that accelerated clotting time in plasma also showed stronger biofilm formation. *S. epidermidis* mutants, deficient in polysaccharide intercellular adhesin (PIA) or the autolysin AtlE were impaired in biofilm formation and had prolonged clotting times compared to their wild type. The wild-type strain induced activation of factor XII and plasma kallikrein, accelerated intrinsic coagulation, and degraded high-molecular-weight kininogen, effects absent in the AtlE and PIA mutants. Notably, DNase I treatment of the wild-type strain prolonged intrinsic coagulation time and prevented high-molecular-weight kininogen degradation, identifying bacterial extracellular DNA as a possible central driver of contact activation. The D5-derived peptide HKH20, previously shown to inhibit contact activation, also reduced *S. epidermidis*–induced activation of contact factors. In plasma, HKH20 decreased the formation and size of bacterial aggregates and altered biofilm architecture by modulating fibrin network formation. Together, these findings identify extracellular DNA and PIA as biofilm-relevant bacterial factors that are linked to contact system activation and intrinsic coagulation in plasma, highlighting an unexpected functional interface between biofilm matrix components and host plasma defense mechanisms.

## 1 Introduction

*Staphylococcus epidermidis* is a skin commensal and an opportunistic pathogen that frequently causes infections associated with indwelling medical devices such as catheters, prosthetic joints, and vascular implants. A hallmark of these infections is the ability of *S. epidermidis* to form biofilms—structured bacterial communities embedded in a self-produced extracellular matrix that protect the bacteria from immune clearance and antibiotic treatment (Severn and Horswill, 2022). Polysaccharide intercellular adhesin (PIA) and the autolysin AtlE are key factors in the early stages of biofilm formation by *S. epidermidis* (Otto, 2009). PIA promotes intercellular aggregation through exopolysaccharide matrix production and binding to fibronectin (Heilmann et al., 1996; Christner et al., 2010; Büttner et al., 2020), while AtlE mediates initial adhesion to abiotic surfaces due to the release of extracellular DNA (eDNA), which supports biofilm formation of the surviving population (Heilmann et al., 1997; Qin et al., 2007).

In parallel, foreign surfaces and certain bacterial pathogens are known to trigger activation of the human contact system (Oehmcke-Hecht and Köhler, 2018), a proteolytic cascade comprising Factor XII (FXII), plasma kallikrein (PK), and high molecular weight kininogen (HK). Activation of this system initiates inflammation through the kallikrein kinin pathway and intrinsic coagulation due to activation of factor XI (FXI). While the role of the contact system in biomaterial-induced thrombosis is well established (Jaffer et al., 2015; Shamanaev et al., 2022), its interaction with *S. epidermidis* or its role in biofilm formation is not defined.

In this study, we investigated the relationship between biofilm formation and coagulation activation by *S. epidermidis*. We used clinical isolates and isogenic mutant strains to dissect how biofilm formation capacity affects clotting responses, FXIIa/PK activity, and HK degradation. We also examined the modulatory potential of the synthetic peptide HKH20 on these interactions. HKH20 is derived from domain 5 of HK and has previously been shown to inhibit contact activation on bacterial surfaces (Oehmcke et al., 2009b), making it a useful tool to probe the contribution of contact system activation in this model. Our findings provide new insight into the host-pathogen interface and suggest that targeting the coagulation–biofilm axis may represent a novel strategy for preventing *S. epidermidis*–associated infections on biomaterial.

## 2 Materials and methods

### 2.1 Material

Citrated plasma from three healthy donors was obtained from the Universitätsmedizin Rostock Institut für Transfusionsmedizin. The plasma was pooled before the experiments. PK-deficient plasma was purchased from George King Bio-Medical (United States). HKH20 (HKHGHGHGKHKNKGKKNGKH) was produced by Peps4LS GmbH (Heidelberg, Germany). In recent studies (Oehmcke et al., 2009b) and in the present study, 50 µM HKH20 produced maximal inhibition of contact activation while not affecting extrinsic coagulation and was therefore used in the majority of assays.

### 2.2 Bacterial strains and culture conditions

*S. epidermidis* wild-type (WT strain 1457, biofilm, icaADBC- and PIA-positive strain) and its PIA mutant strain (*S. epidermidis* 1457-M10 1457icaA:Tn917, PIA- and biofilm-negative), the PIA-complemented strain (PIA compl., 1457-M10 containing plasmid pTXicaADBC, expressing icaADBC under the control of a xylose-inducible promoter), and the AtlE mutant strain (*S. epidermidis* mutant strain 1457atlE:Tn917, *atlE-* and biofilm-negative) have been described elsewhere (Heilmann et al., 1997; Mack et al., 2000, Mack et al., 2001). Bacteria were grown in BHI (if not otherwise indicated) at 37°C with 5% CO_2_. If the PIA compl. strain was used; BHI was supplemented with 4% xylose.

A collection of 50 clinical *S. epidermidis* strains from invasive infections was cultured on Columbia blood agar and stored in microbank vials at −80°C (Ethical statement: A 2024-0185). For the experiments, clinical isolates were cultivated in BHI medium at 37°C with 5% CO_2_.

### 2.3 Clotting assays

Activated partial thromboplastin time (aPTT), prothrombin time (PT), and thrombin clotting time (TCT) in the presence of HKH20 were determined in a coagulometer (Amelung). For aPTT, plasma was activated with Dapptin reagent (Technoclone) and recalcified by addition of CaCl_2_ (25 mM). For TCT, plasma was activated with thrombin reagent (Technoclone), and for PT, plasma was activated with Technoplastin HIS (PT reagent, Technoclone), and clotting times were measured.

### 2.4 Clotting with nanoparticles or catheter segments

Nanoparticles (Resovist, iron oxide particles, Schering, Germany) at 90 µg/ml were added to recalcified plasma or to recalcified plasma containing 45 µM HKH20, and clotting time was measured in a coagulometer. Catheters (3 lumen catheter kit, 7.5 Fr – 20 cm, Vygon, Germany) were cut into 5 mm segments and placed in the cuvette of the coagulometer. Plasma or plasma containing HKH20 (50 µM) was added and incubated for 60 s. To induce coagulation, CaCl_2_ (25 mM) was added.

### 2.5 Clotting assays with bacteria

Clotting of bacteria was performed from an overnight culture that was adjusted to 10^10^ CFU/ml in HEPES buffer, added to plasma (1:1), and incubated for 30 min. Plasma was recalcified by the addition of CaCl_2_ (25 mM) and clotting time was measured in the coagulometer. For aPTT, plasma was activated with Dapptin reagent (Technoclone) and recalcified by addition of CaCl_2_ (25 mM). For TCT, plasma was activated with thrombin reagent (Technoclone), and for PT, plasma was activated with Technoplastin HIS (PT reagent, Technoclone), and clotting times were measured.

### 2.6 Turbidimetric assay

HKH20 stock solution (455 µM) was serially diluted in Aqua dest. to obtain the indicated concentrations. For each condition, 100 µl of either PBS (control) or HKH20 dilution was mixed with 343 µl of pooled plasma and tPa (2.9 nM, Sigma-Aldrich Chemie GmbH, Steinheim, Germany). Aliquots were transferred into individual wells of a 96-well plate (polyurethan), and CaCl_2_ (15 mM) was added to each well. Accordingly, the kinetic measurement was performed at a wavelength of 340 nm every 15 s for 3h. The analysis of generated data was carried out by the ClotlysisCL_2019 application from Dr. Colin Longstaff (https://drclongstaff.shinyapps.io/clotlysisCL_2019/) to obtain time to 50% clotting (Longstaff and fibrinolysis, 2017).

### 2.7 Biofilm formation in BHI

Bacteria from an overnight culture, grown in BHI medium, were adjusted to an optical density at 600 nm of 0.5 in fresh BHI medium (+4% xylose) and then diluted 1:10. 0.2 ml of the bacterial suspension per well was pipetted into a 96-well plate and incubated at 37°C and 5% CO_2_ for 24h, 48h, or 72h. Afterwards, the plates were washed, and the bacteria were stained with crystal violet. Absorption was determined at 570 nm.

### 2.8 Biofilm formation in human plasma-like medium or plasma

Bacteria (100 µl) from an overnight culture were transferred into the wells of a washed to remove non 96-well plate and incubated for 1h at 37°C and 5% CO_2_. The plates were -adherent bacteria, filled with human plasma-like medium (HPLM) or plasma (supplemented with HKH20 at the indicated concentration), and incubated at 37°C, 5% CO_2_ for 24h. Afterwards, the plates were washed, and the bacteria were stained with crystal violet. Absorption was determined at 570 nm.

### 2.9 Monitoring bacterial aggregates

*S. epidermidis* WT was incubated with plasma or with plasma supplemented with HKH20 (50 µM) for 2h at 37°C. Following incubation, bacterial smears were prepared and Gram-stained. The smears were examined using an Olympus SC50 microscope (Olympus, Hamburg, Germany), and 10 images were taken per smear. Image analysis was performed using ImageJ (National Institutes of Health, Bethesda, USA). A minimum aggregate size of 500-pixel units was defined, and both the number and size of aggregates were recorded.

### 2.10 PK/FXIIa or FXIa activity in plasma

Bacteria from an overnight culture were set to 1 × 10^10^ CFU/ml in 50 mM Tris buffer and added to 1:10 diluted plasma (1:1). Plasma was diluted 1:10 to reduce background activity and optimize detection of substrate cleavage in the chromogenic assay. Buffer instead of plasma was used as a control. Autoactivation controls (plasma + buffer) were included in each assay. As absorbance values remained consistently below 0.05, these controls are not shown. The WT was also incubated in the presence of 25 µM HKH20 or 4 units DNase I (Biozym, Hessisch Oldendorf, Germany). Substrate S-2302 (specific for FXIIa/PK, Cryopep, Montpellier, France) or S-2366 (specific for FXIa/APC, Cryopep) was added to all samples and incubated for 35 min at 37°C. Bacteria were removed by centrifugation, and an endpoint measurement of the supernatants was performed at 405 nm in the spectramax.

### 2.11 Western blot analysis of HK degradation in plasma

Plasma was mixed in a 1:1 ratio with bacteria (1 × 10^10^ CFU/ml) or the FXII activator Dapptin (Technoclone) and incubated at 37 °C for indicated time points. After incubation, the samples were briefly centrifuged, and the supernatants were collected in SDS-PAGE buffer. Plasma (1 µl) was applied to each lane and fractionated on 4%–12% gradient SDS-polyacrylamide gels followed by immunoblotting. The membrane was stained with an antibody against HK (Biozol, Eching, DE) and detected with a Donkey-anti-goat-IRDye (LI-COR Biosciences, Lincoln, NE, USA). Blot was imaged on Odyssey Imager (LI-COR).

### 2.12 Scanning electron microscopy

An overnight culture of *S. epidermidis* WT was prepared in 10 ml HPLM and incubated at 37°C and 5% CO_2_. The next day, 1 ml of the bacteria was added to a 24-well plate, equipped with a sterile plastic coverslip, and incubated for 1h at 37°C and 5% CO_2_. Non-adherent bacteria were removed and the wells filled with BHI, plasma, or plasma with HKH20 (50 µM). For the controls, wells were filled with plasma or plasma with HKH20 (50 µM) and incubated for 24h. Biofilms were allowed to develop for 24h, fixed and prepared as described before (Isenring et al., 2017; Köhler et al., 2019). Samples were visualized with a MERLIN VP Compact field emission scanning electron microscope (Carl Zeiss, Jena, Germany) operated at the Electron Microscopy Center (EMZ).

## 3 Results

### 3.1 Clinical strain variation reveals an inverse relationship between clotting time and biofilm formation

**Figure 1:**
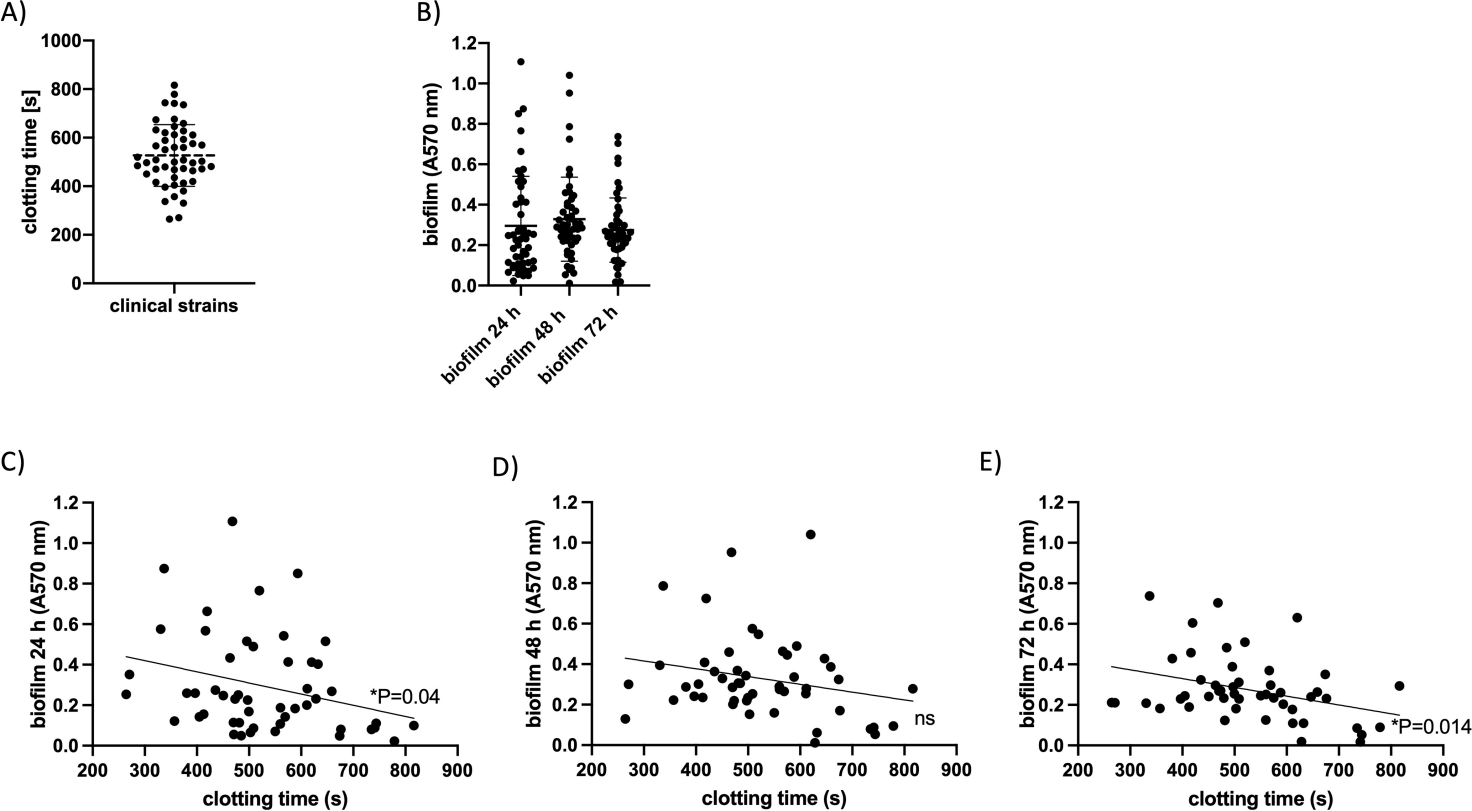
Clinical strain variation reveals an inverse relationship between clotting time and biofilm formation. **(A)** Bacteria were adjusted to 10^10^ CFU/ml in HEPES buffer, added to recalcified plasma, and the time until clot formation was measured in a coagulometer. *n* = 3–4/strain; **(B)** Biofilm formation was measured after bacteria from an overnight culture were adjusted to an optical density at 600 nm of 0.5 in fresh BHI medium (+ 4% xylose) and then diluted 1:10. 0.2 ml of the bacterial suspension per well was pipetted into a 96-well plate and incubated at 37°C and 5% CO_2_ for 24h, 48h, or 72h. Afterwards, the plates were washed, and the bacteria were stained with crystal violet. Absorption was determined at 570 nm. **(C, D)** Simple linear regression using clotting time (see **(A)**) as the independent variable and biofilm absorbance (OD_570_) as the dependent variable.

First, we explored the ability of *S. epidermidis* to induce coagulation in recalcified plasma and how this might correlate with biofilm formation capacity. Thus, we analyzed a collection of clinical *S. epidermidis* isolates (*n* = 50) obtained from bloodstream infections and indwelling medical devices. Overnight cultures of each strain were set to 10^10^ CFU/ml and incubated in recalcified human plasma. The clotting time—defined as the time until fibrin clot formation—was measured in a coagulometer. Clotting times ranged widely among the strains, from approximately 230–814 s (**Figure 1A**), reflecting significant variation in the ability to trigger coagulation. In parallel, we assessed biofilm formation using a crystal violet staining assay after 24h, 48h, and 72h incubation (**Figure 1B**). To explore the relationship between pro-coagulant activity and biofilm formation, we performed simple linear regression, using clotting time as the independent variable and biofilm absorbance (OD_570_) as the dependent variable (**Figures 1C–E**). The analysis revealed a significant negative association, indicating that strains capable of faster coagulation also tended to form more robust biofilms. This inverse relationship was significant at 24h and 72h time points (**Figures 1C, E**), suggesting a stable, strain-intrinsic link between coagulation-triggering properties and biofilm-forming capacity. The correlation between biofilm biomass and clotting time was less pronounced at the intermediate 48h time point, which may reflect dynamic changes in biofilm architecture and matrix composition during biofilm maturation. However, these findings suggest that phenotypes promoting faster coagulation in plasma may coincide with traits that enhance biofilm formation, even in plasma-free conditions.

### 3.2 Biofilm-deficient *S. epidermidis* mutants exhibit prolonged clotting times in plasma

**Figure 2:**
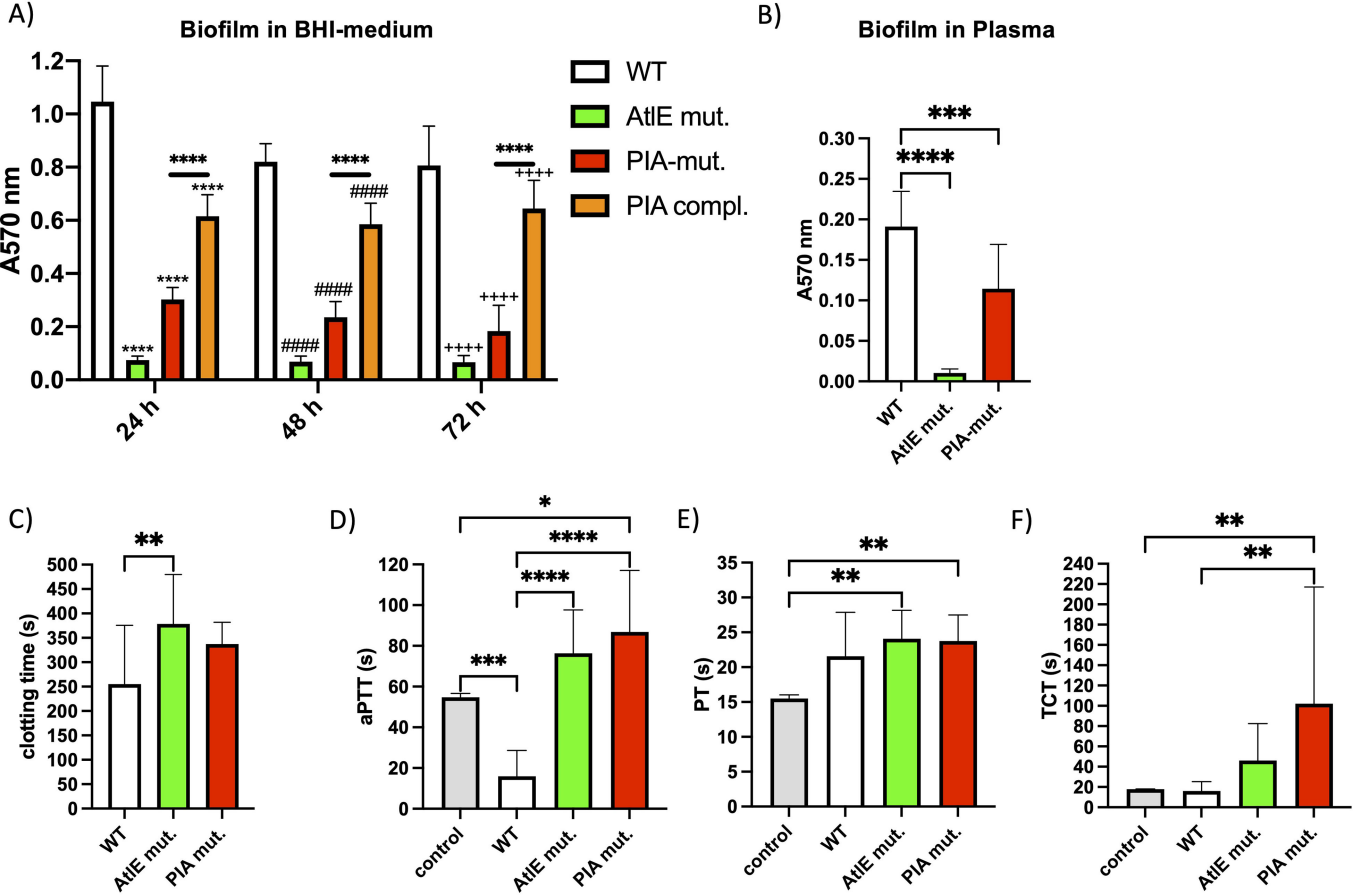
PIA and AtlE deficiency impaired biofilm formation and clotting times. **(A)** Bacteria from an overnight culture were adjusted to an optical density at 600 nm of 0.5 in fresh BHI medium (+4% xylose) and then diluted 1:10. 0.2 ml of the bacterial suspension per well was pipetted into a 96-well plate and incubated at 37°C and 5% CO_2_ for 24h, 48h, or 72h. Afterwards, the plates were washed, and the bacteria were stained with crystal violet. Absorption was determined at 570 nm. (*n* ≥ 4), ****, ^####^, or ^++++^*P* < 0.0001 tested by ordinary two-way analysis of variance (ANOVA) followed by Tukey’s multiple-comparison post-test. *applies in relation to the WT 24h, ^#^applies in relation to the WT 48h, ^+^applies in relation to the WT 72h. **(B)** 100 µl of bacteria from an overnight culture were transferred into the wells of a 96-well plate, and incubated for 1h at 37°C and 5% CO_2_. The plates were washed to remove non-adherent bacteria, filled with plasma, and incubated at 37°C, 5% CO_2_ for 24h. Afterwards, the plates were washed, and the bacteria were stained with crystal violet. Absorption was determined at 570 nm. (*n* ≥ 4), ****P < 0.0001, ***P < 0.001, tested by ordinary one-way ANOVA followed by Tukey’s multiple-comparison post-test. **(C)** Bacteria from an overnight culture were adjusted to 1 × 10^10^ CFU/ml in HEPES buffer, added to plasma, and incubated for 30 min. Plasma was recalcified, and clotting time was measured. *n* ≥ 4; **P < 0.01, tested by ordinary-one-way ANOVA followed by Šídák’s-multiple comparisons test. Error bars = SD. **(D–F)** Bacteria from an overnight culture were adjusted to 1 × 10^10^ CFU/ml in HEPES buffer, added to plasma, and incubated for 30 min. In the control, HEPES buffer instead of bacteria was used. For aPTT **(D)** the bacteria-plasma mix was activated with Dapptin reagent and recalcified. For PT **(E)** the bacteria-plasma mix was activated with prothrombin time reagent, and clotting time was measured. For TCT **(F)**, the bacteria-plasma mix was activated with thrombin. *n* ≥ 4; *P < 0.05, **P < 0.01, ***P < 0.001, ****P < 0.0001 tested by one-way ANOWA or Kruskal–Wallis test, followed by Šídák’s or Dunn`s multiple-comparison post-test. Error bars = SD.

The inverse relationship between clotting time and biofilm formation among clinical *S. epidermidis* isolates suggested that bacterial factors promoting biofilm formation may also contribute to procoagulant activity. To investigate this mechanistic link, we analyzed a biofilm-proficient *S. epidermidis* wild type and its mutants, lacking the biofilm-associated genes PIA and AtlE (Mack et al., 2001). We confirmed that both the AtlE and PIA mutant strains had significantly reduced biofilm formation compared to the WT strain (**Figure 2A**). The PIA-complemented strain showed significantly increased biomass compared to the PIA mutant strain (**Figure 2A**), supporting the important role for PIA in biofilm formation. To assess early surface interactions and subsequent biofilm formation in human plasma, bacteria were first incubated for 1h in wells of a polyurethan 96-well plate, to allow initial adherence. After washing to remove non-adherent bacteria, wells were filled with human plasma, and biofilms were allowed to develop for 24h, followed by crystal violet (CV) staining. Although biofilm formation was significantly reduced under these conditions, both the AtlE and PIA mutant strains also showed significantly reduced biofilm formation compared to the WT strain (**Figure 2B**). As the PIA-complemented strain requires 4% xylose to induce PIA production, which could not be added in this plasma assay, the PIA compl. strain was not tested. However, we confirmed the deficiencies of the AtlE and PIA mutants in biofilm production and surface adhesion.

We then evaluated the impact of these mutations on plasma clotting times. The AtlE mutant strain showed a prolonged time to clot formation (clotting time), compared to the WT strain (**Figure 2C**). To better characterize this effect, standard clotting assays were performed. The activated partial thromboplastin time (aPTT), as a test for intrinsic coagulation, was significantly shortened when plasma was incubated with WT bacteria (**Figure 2D**), indicating contact system activation. Incubation with both mutants significantly prolonged aPTT (**Figure 2D**). The prothrombin time (PT), as a marker for extrinsic coagulation, was not changed when incubated with the WT; however, incubation with both mutants significantly prolonged PT compared to the control (**Figure 2E**). The thrombin clotting time (TCT) was significantly prolonged only by the PIA mutant (**Figure 2F**). Taken together, this pattern indicates a specific interference with the intrinsic coagulation pathway by *S. epidermidis*, with both AtlE and PIA being involved.

### 3.3 *S. epidermidis*–induced PK/FXIIa, FXIa activity, and degradation of HK in plasma

**Figure 3:**
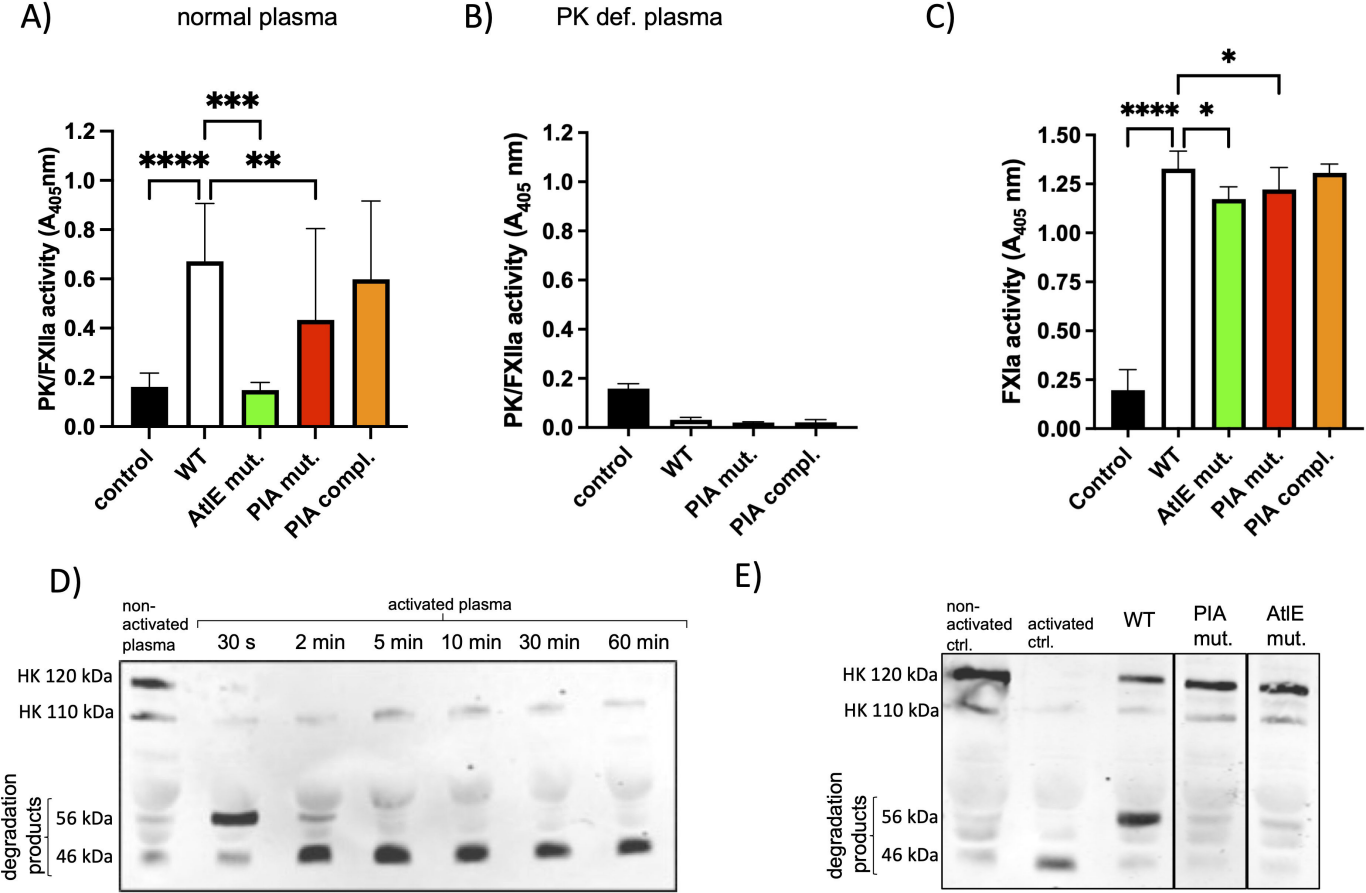
*S. epidermidis* induced FXII/PK, FXI activity, and partial degradation of HK in plasma, that was decreased by biofilm-deficient mutants. Bacteria from an overnight culture were set to 1 × 10^10^ CFU/ml and added to 1:10 diluted normal plasma **(A, C)** or PK-def. plasma **(B)**. Bacteria added to buffer instead of plasma is shown as negative control (A–C, control). Substrate S-2302 (specific for FXIIa/PK, A, B) or S-2366 (for APC/FXIa, C) was added to all samples and incubated for 35 min at 37°C. Bacteria were removed by centrifugation and an endpoint measurement of the supernatants was performed at 405 nm. *n* ≥ 4. ****P < 0.0001, ***P < 0.001, **P < 0.01, *P < 0.05, compared to the WT, tested by Kruskal–Wallis test, followed by Dunn`s multiple-comparison post-test. Error bars = SD. **(D)** Plasma was mixed 1:1 ratio with PBS (non-activated) or the FXII activator Dapptin (activated plasma) and incubated at 37 °C for the indicated time points. After incubation, the samples were briefly centrifuged, and the supernatants were collected in SDS-PAGE buffer. 1 µl plasma was applied to each lane. The membrane was stained with an antibody against HK. **(E)** Bacteria (1 × 10^10^ CFU/ml) were mixed with plasma (1:1 ratio) and incubated for 60 min at 37°C and 600 rpm. For the non-activated control (non-activated ctrl.) PBS was mixed with plasma (ratio 1:1) and for the activated control plasma was mixed with Dapttin (1:1). After incubation, the samples were centrifuged, and the plasma supernatants were collected and applied to an SDS page. The membrane was stained with a polyclonal antibody against HK.

To explore the mechanism behind intrinsic coagulation activation by *S. epidermidis*, we examined whether the bacteria activate PK/FXII and FXI and degrade HK. Using a chromogenic substrate assay, we observed robust PK/FXIIa activity in plasma incubated with WT *S. epidermidis*. This activation was significantly reduced when incubated with the AtlE and PIA mutants, although the reduction of PK/FXIIa activity in the AtLE mutant was significantly more prominent than in the PIA mutant (**Figure 3A**). These findings indicate that the presence of AtlE and PIA contributes to activation of the contact system factors PK/FXII. Importantly, no substrate cleavage occurred when bacteria were incubated in buffer (control) or in PK-deficient plasma (**Figure 3B**), confirming that the measured activity was specific to PK. To determine whether this activation propagates downstream within the intrinsic coagulation pathway, we next assessed FXI activation using a chromogenic FXIa substrate. Plasma incubated with WT *S. epidermidis* showed strong substrate cleavage, whereas both the AtlE- and PIA-deficient mutants exhibited significantly reduced activity. Notably, FXI-associated activity was restored to WT levels in the PIA-complemented strain (**Figure 3C**), further supporting the role of biofilm-associated factors in promoting activation of intrinsic coagulation downstream of the contact system.

To assess whether PK activity leads to HK degradation, plasma was incubated with *S. epidermidis* strains or Dapptin (an FXII activator) for up to 60 minutes and analyzed by Western blot using anti-HK antibodies. In control plasma, intact HK appeared at 120 kDa, with a faint intermediate at ~110 kDa (**Figure 3D**, non-activated plasma). Dapptin treatment caused HK cleavage within 30 s, resulting in disappearance of the 120 kDa band and appearance of 56 and 46 kDa fragments (**Figure 3D**, activated plasma), consistent with two-chain HK formation (Herwald et al., 2001). Incubation with *S. epidermidis* WT led to a reduced 120 kDa HK band and prominent appearance of the 56 kDa fragment, indicating HK cleavage (**Figure 3E**, WT). This intermediate form is consistent with early HK processing seen in contact system activation (Mori and Nagasa WA, 1981). No HK degradation was observed with the PIA or AtlE mutant (**Figure 3E**). Similarly, no cleavage occurred in PK-deficient plasma (not shown), confirming that HK degradation was PK-dependent. These findings demonstrate that *S. epidermidis* can induce degradation of HK in plasma. Moreover, AtlE seems to be mainly involved in the induction of PK/FXIIa activity.

**Figure 4:**
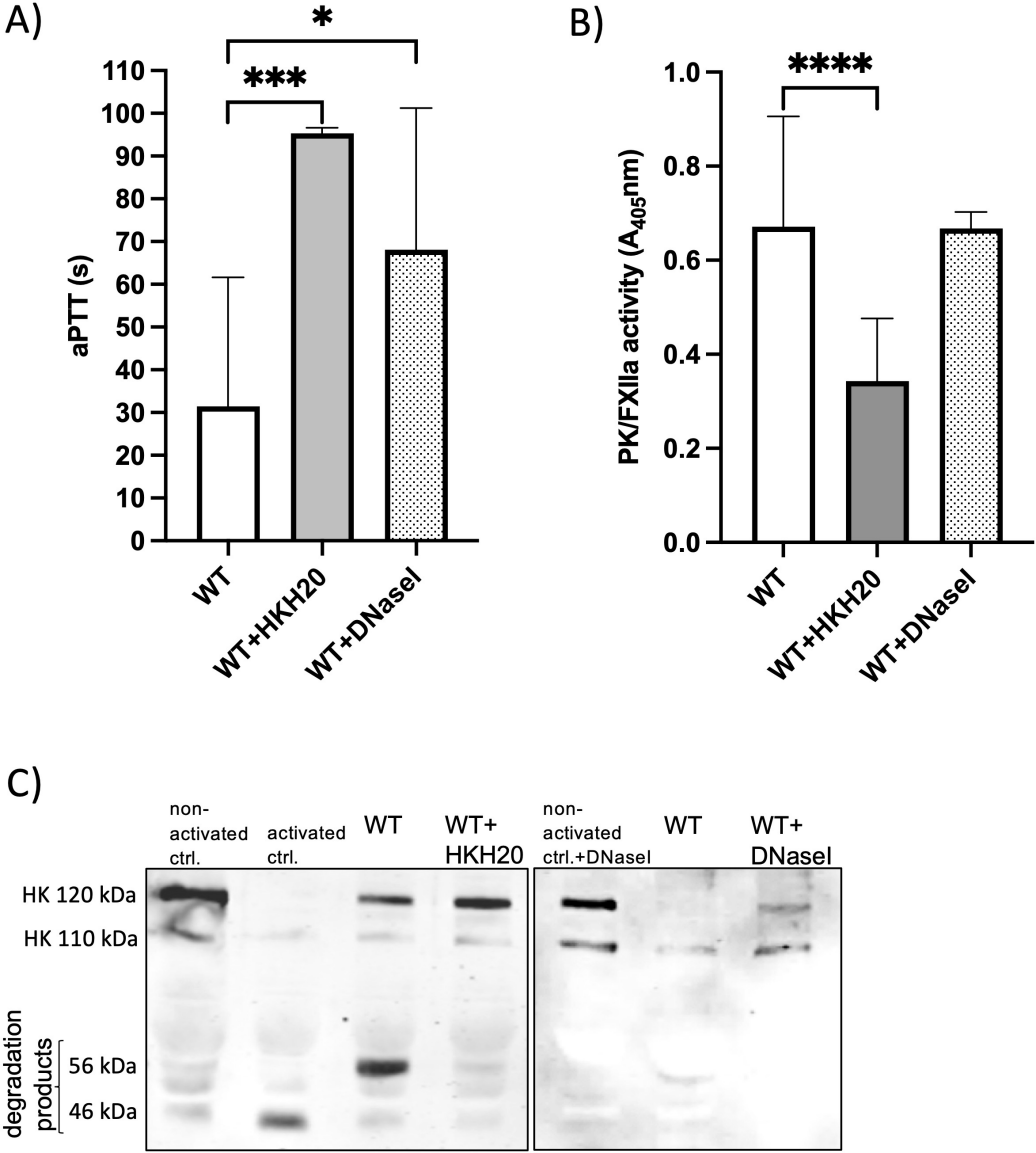
Treatment with the peptide HKH20 or with DNaseI prolonged aPTT and prevented degradation of HK in plasma. **(A)** Wild-type (WT) bacteria from an overnight culture were adjusted to 1 × 10^10^ CFU/ml in HEPES buffer, added to plasma with buffer (WT) or HKH20 (25 µM) or DNaseI (2 units), and incubated for 30 min. For aPTT, the bacteria–plasma mix was activated by Dapptin, recalcified, and aPTT was measured. *n* ≥ 4; ***P < 0.001, *P < 0.05 tested by ordinary one-way analysis of variance (ANOVA) followed by Dunnett`s multiple comparisons test. Error bars = SD. **(B)** Bacteria from an overnight culture were set to 1 × 10^10^ CFU/ml and added to 1:10 diluted plasma with buffer (WT) or HKH20 (25 µM, WT+HKH20) or DNaseI (2 units, WT+DNaseI). Substrate S-2302 (specific for PK) was added to all samples and incubated for 35 min at 37°C. Bacteria were removed by centrifugation and an endpoint measurement of the supernatants was performed at 405 nm. *n* ≥ 4. ****P < 0.0001, compared to the WT, tested by ordinary one-way ANOVA followed by Tukey’s multiple-comparison post-test. Error bars = SD. C) Plasma was mixed 1:1 ratio with PBS (non-activated ctrl.), or Dapptin (activated ctrl.), or 10^10^ CFU/ml bacteria with 45 µM HKH20 (WT+HKH20) or 4 Units DNaseI (WT+DNAseI) or equal volume of PBS (WT), and incubated for 60 min at 37°C and 600 rpm. After incubation, the samples were briefly centrifuged, the supernatants collected in SDS-page buffer and applied to an SDS page. The membrane was stained with an antibody against HK.

As AtlE is associated with the release of extracellular DNA (eDNA), which might provide a surface for contact activation, we tested whether incubation with DNaseI influences the aPTT. Additionally, we tested the aPTT in the presence of HKH20, a peptide derived from domain 5 of HK, that blocks contact activation by *Streptococcus pyogenes* (Oehmcke et al., 2009b). Both the addition of the peptide HKH20 and that of DNase I prolonged the aPTT significantly (**Figure 4A**), although the aPTT after the addition of DNase I was very variable.

PK/FXIIa activity in the presence of HKH20 was also significantly reduced, whereby DNase I had no influence in this substrate assay (**Figure 4B**). However, both HKH20 and DNase I treatments preserved intact HK and prevented cleavage by WT bacteria (**Figure 4C**).

To assess whether *S. epidermidis* directly binds or adsorbs host proteins known to participate in coagulation and contact activation, we incubated the WT strain with plasma and probed for adsorption of fibrinogen (**Supplementary Figure 1**), plasminogen, or HK (not shown). In contrast to *S. pyogenes* and *S. aureus* (**Supplementary Figure 1A**), no significant binding of these proteins to the bacterial surface of *S. epidermidis* was detected by immunoblotting. To further assess whether other *S. epidermidis strains* adsorb relevant plasma proteins under the conditions used in this study, six clinical isolates were incubated in human plasma and analyzed for associated fibrinogen or PK. No detectable adsorption of either protein was observed in any of the strains tested (**Supplementary Figures 1B**, **S1C**). These findings suggest that the observed activation of the contact system by *S. epidermidis* is not dependent on specific surface acquisition of these plasma proteins.

### 3.4 HKH20 impacts the formation of bacterial aggregates in plasma

**Figure 5:**
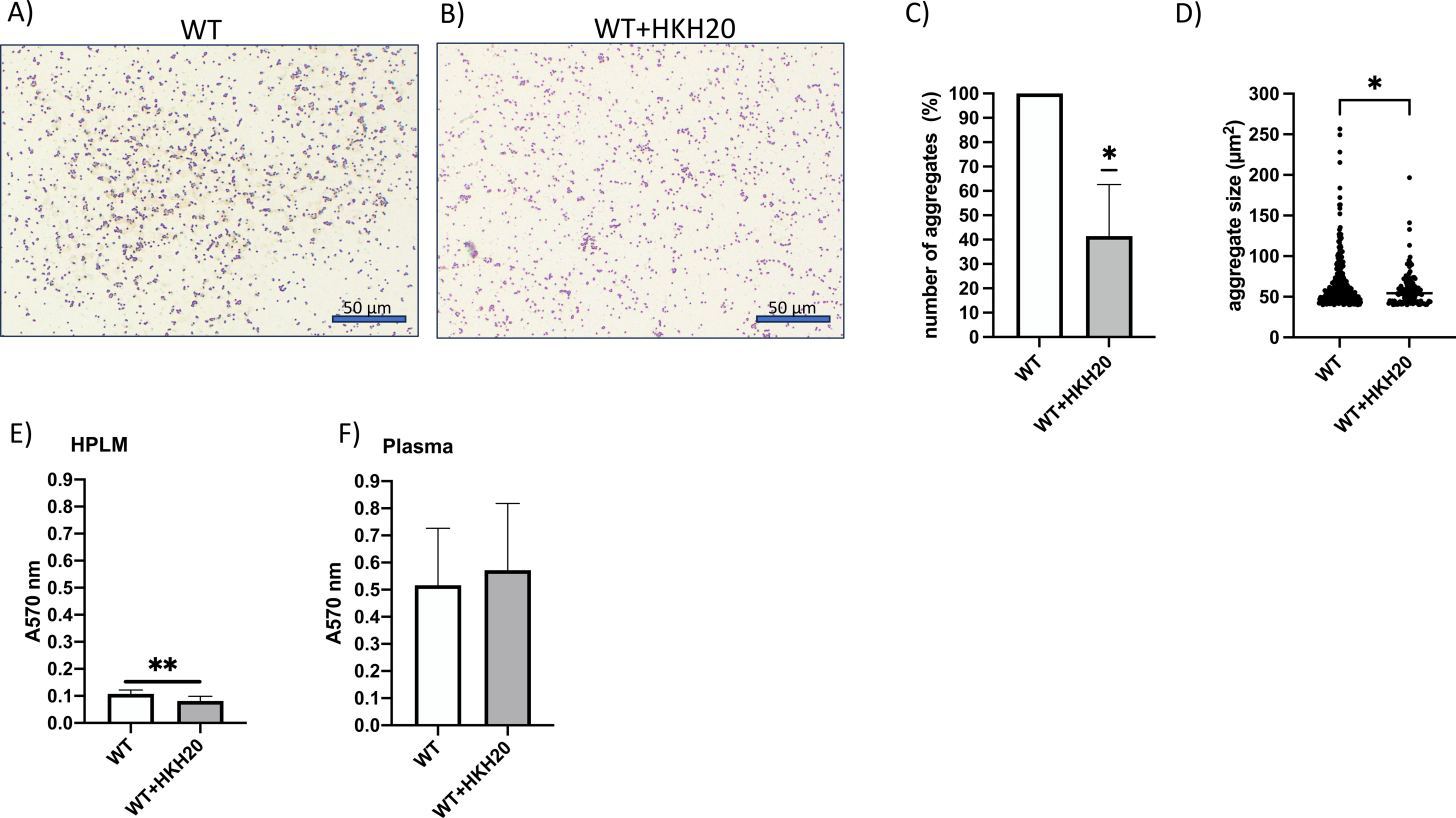
HKH20 impacts formation of bacterial aggregates but not biofilm formation in plasma. **(A, B)***S. epidermidis* WT was incubated with plasma or with plasma supplemented with HKH20 (50 µM) for 2h at 37°C. Following incubation, bacterial smears were prepared, and Gram-stained. Bars represent 50 µm. **(C)** The smears were examined by microscopy, and bacterial aggregates were counted. The absolute number of aggregates larger than 40 µm² was normalized to the control (WT in plasma without HKH20). Data from three independent experiments were pooled. *P = 0.0408, tested by one sample t-test. **(D)** Shown is the aggregate size (including only aggregates larger than 40 µm²) from three independent experiments. *P = 0.0331, tested by Man Whitney test. **(E, F)** Bacteria from overnight culture were incubated for 1h in wells of a polyurethane 96-well plate, to allow initial adherence. After washing to remove non-adherent bacteria, wells were filled with HPLM **(E)** or full plasma **(F)** with or without HKH20 (50 µM). Biofilms were allowed to develop for 24h, followed by crystal violet (CV) staining. **P < 0.01, tested by unpaired t-test.

Given that HKH20 inhibited contact activation induced by WT *S. epidermidis*, we next examined whether the peptide also affects formation of bacterial aggregates and biofilm formation in plasma. For this, WT *S. epidermidis* was incubated with plasma or with plasma supplemented with HKH20 for 2h at 37°C. Following incubation, bacterial smears were prepared, Gram-stained, and bacterial aggregates larger than 40 µm² were quantified (**Figures 5A, B**). This analysis revealed that incubation with HKH20 led to a significant reduction in the number of large bacterial aggregates (**Figure 5C**), as well as a decrease in mean aggregate size (**Figure 5D**), compared to bacteria incubated in plasma alone. These findings suggest that HKH20 interferes with bacterial aggregation in plasma, potentially contributing to its anti-coagulative effects.

To assess a potential effect of HKH20 on biofilm formation, bacteria were incubated for 1h in wells of a polyurethan 96-well plate, to allow initial adherence. After washing to remove non-adherent bacteria, wells were filled with either human plasma-like medium (HPLM) or full human plasma, with or without HKH20. Biofilms were allowed to develop for 24h, followed by crystal violet (CV) staining. In HPLM, *S. epidermidis* formed limited biofilm; however, the presence of HKH20 significantly reduced the biomass further (**Figure 5E**). In contrast, when grown in full plasma, HKH20 had no additional effect on biomass under these conditions (**Figure 5F**).

### 3.5 HKH20 alters biofilm architecture in plasma

**Figure 6:**
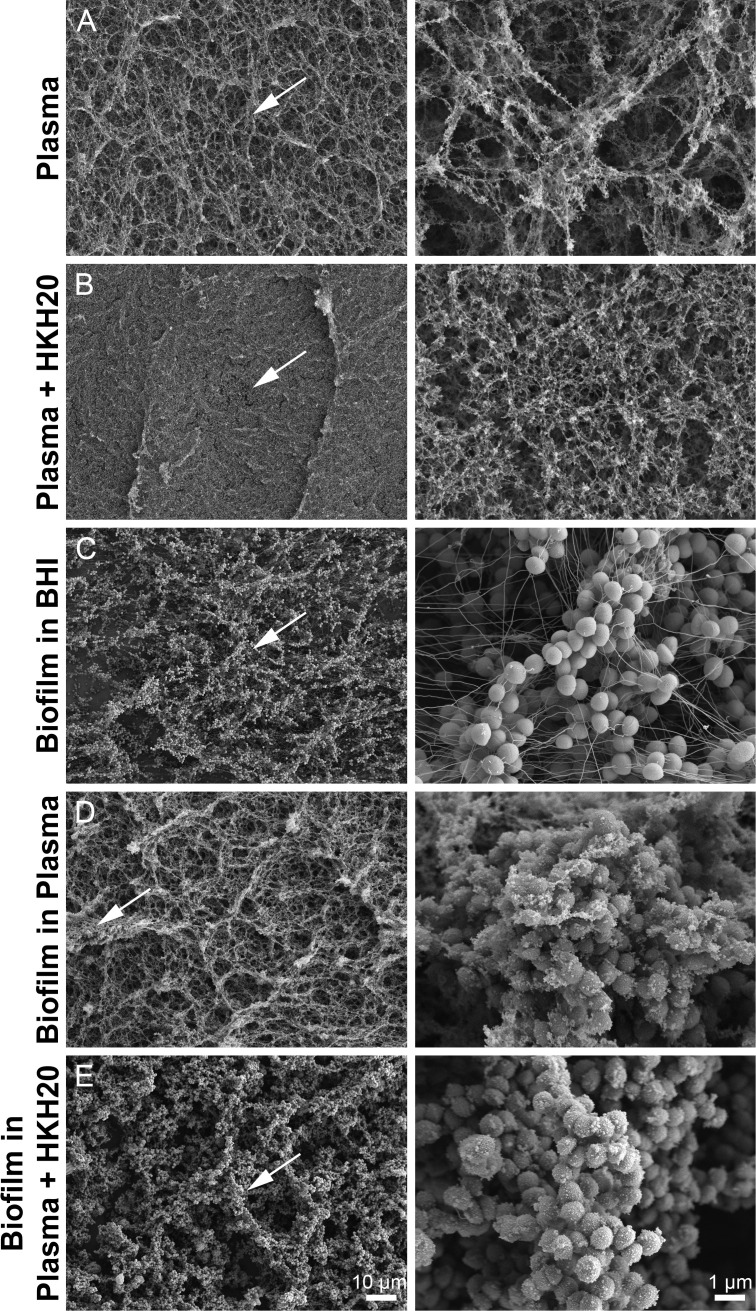
HKH20 modulates fibrin network formation and changed biofilm architecture of *S. epidermidis* in plasma. **(A, B)** Plasma or plasma supplemented with HKH20 (50 µM) was incubated for 24h on polyurethane surfaces. **(C–E)** An overnight culture of *S. epidermidis* WT was prepared in 10 ml HPLM and incubated at 37°C and 5% CO_2_. The next day, 1 ml of the bacteria was added to a 24-well plate, equipped with a sterile plastic coverslip, and incubated for 1h at 37°C and 5% CO_2_. Non-adherent bacteria were removed and the wells filled with BHI **(C)**, plasma **(D)**, or plasma with HKH20 (50 µM) **(E)**. Biofilms were allowed to develop for 24h, fixed, and analyzed by scanning electron microscopy. Low magnification overview images (x1000) are shown in the left column. White arrows indicate the area selected for the corresponding higher magnifications (×10.000) shown in the right column. Scale bars are 1 µm and 10 µm, respectively.

The aggregation assay shown in **Figure 5** reflects early bacterial aggregation in plasma after short-term incubation (2h). To further investigate the impact of HKH20 on surface and biofilm structure, we performed SEM (**Figure 6**). When polyurethane surfaces were incubated with plasma, a dense fibrin network was observed, covering much of the material (**Figure 6A**, Plasma). In contrast, surfaces exposed to plasma containing HKH20 showed a less dense fibrin network composed of finer fibers (**Figure 6B**, Plasma+HKH20), further supporting the modulatory effects of the peptide on fibrin architecture and contact activation.

To investigate biofilm structures, bacteria were allowed to adhere to a plastic coverslip for 1h, non-adherent bacteria were removed, and biofilms were subsequently grown for 24h in either BHI medium, human plasma, or plasma supplemented with HKH20. In BHI medium, *S. epidermidis* formed dense, multilayered biofilms. At higher magnification, we observed smooth bacterial surfaces and long filamentous structures connecting neighboring cells, consistent with extracellular matrix structures associated with the biofilm (**Figure 6C**, biofilm in BHI). In contrast, biofilms grown in plasma displayed markedly different morphology. A prominent fibrin network was observed covering the surface, with bacteria appearing as clusters interspersed within the fibrin mesh (**Figure 6D**, biofilm in plasma). At higher resolution, aggregates were visible on the surfaces of the bacteria incubated in plasma. These results suggest that plasma components were integrated in biofilm formation and altered the surface environment, with bacteria appearing embedded within the fibrin network. When biofilms were formed in plasma supplemented with HKH20 (**Figure 6E**, biofilm in plasma+HKH20), the fibrin network was largely absent, and fibrin fibers were not readily detectable. Instead, bacteria formed structured aggregates more reminiscent of the BHI-grown biofilm. However, at higher magnification, the bacterial surfaces appeared rough and irregular, potentially due to altered surface interactions or matrix deposition under conditions of reduced fibrin network formation. These findings indicate that HKH20 reduces the formation of a dense fibrin network and, in addition to its inhibitory effect on contact activation, modulates biofilm architecture on abiotic surfaces.

### 3.6 HKH20 inhibits contact activation and fibrin network formation on artificial surfaces

To further explore the ability of HKH20 to modulate contact activation beyond bacterial contexts, we tested its effects on artificial materials known to trigger coagulation, including polystyrene, catheter segments, and nanoparticles. These experiments were designed to assess whether HKH20 affects surface-induced activation of the contact system and fibrin formation, two processes relevant for thrombosis and device-associated complications.

**Figure 7:**
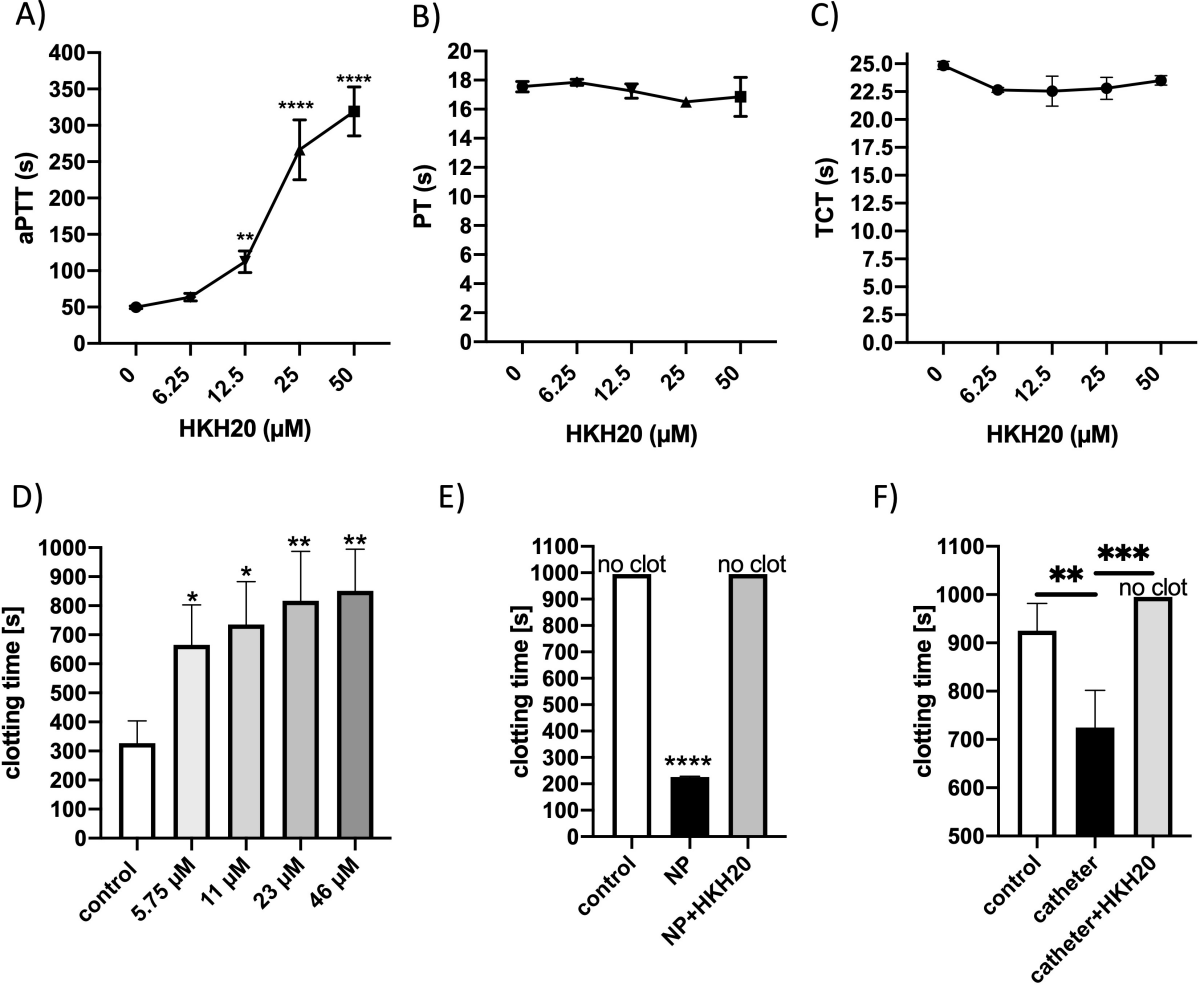
HKH20 prolonged clot formation on different artificial surfaces. Plasma was incubated with increasing amounts of HKH20 and analyzed by the aPTT **(A)**, PT **(B)** or TCT **(C)** test. **P < 0.01, ****P < 0.0001, tested by ordinary one-way analysis of variance (ANOVA) and Dunnett’s multiple comparison posttest. **(D)** Recalcified plasma was incubated with increasing amounts of HKH20 in a 96-well plate (polyurethane), and the time until 50% clotting was determined by a turbidimetric assay. **(E)** Nanoparticles (NP, 90 µg/ml) were added to recalcified plasma, or to recalcified plasma containing 45 µM HKH20, and clotting time was measured in a coagulometer. **(F)** Catheter segments were placed in the cuvette of the coagulometer and plasma or plasma containing HKH20 (50 µM) were incubated for 60 (s) To induce coagulation, CaCl_2_ (25 mM) was added. *P < 0.05, **P < 0.01, ***P < 0.001, tested by ordinary one-way ANOVA and Dunnett’s multiple comparison posttest.

Plasma coagulation assays showed that HKH20 significantly prolonged the aPTT at 12.5 µM (**Figure 7A**), whereas PT and TCT remained unaffected (**Figures 7B, C**), confirming its specific interference with the intrinsic pathway (Oehmcke et al., 2009b). Moreover, HKH20 significantly delayed the onset of clot formation when plasma was exposed to polystyrene surfaces (**Figure 7D**), nanoparticles (**Figure 7E**), or catheter segments (**Figure 7F**).

## 4 Discussion

Our study uncovers a functional relationship between the biofilm-forming capacity of *S. epidermidis* and its ability to activate the human contact system. We show that clinical isolates of *S. epidermidis* exhibit considerable variability in both clotting activation and biofilm formation, and that these two phenotypes are inversely related. Strains with strong biofilm-forming ability tend to induce shorter clotting times, suggesting that surface-related bacterial traits may modulate plasma protein interactions relevant for coagulation.

In our mutant strain analysis, we confirmed that loss of PIA production or AtlE resulted in reduced biofilm formation and, interestingly, prolonged clotting times. The AtlE mutant in particular showed neither activation of PK/FXIIa substrate nor degradation of HK, supporting the notion that AtlE not only contributes to surface adhesion during biofilm formation (Heilmann et al., 1997) but also to conditions that facilitate the activation of the contact system.

Intriguingly, AtlE from *S. epidermidis* is responsible for generation of eDNA as a crucial structural component in the early stages of *S. epidermidis* biofilm. Most of the eDNA in *S. epidermidis* cultures and biofilms is generated through the activity of AtlE (Qin et al., 2007). As especially the AtlE mutant showed no PK/FXIIa activation in our experiments one can speculate that the release of eDNA is mainly responsible. We and others have shown that extracellular eukaryotic DNA, released as neutrophil extracellular traps (NETs), can bind contact factors and trigger their activation (Oehmcke et al., 2009a; Fuchs et al., 2010; Vu et al., 2016), and a similar mechanism probably underlies contact activation by bacterial eDNA. Moreover, we did not observe direct adsorption of key plasma proteins (HK, PK, plasminogen or fibrinogen) to the *S. epidermidis* surface, supporting the idea that activation of the contact system likely results from the exposure of eDNA, rather than specific protein binding or adsorption. This is in contrast to *Staphylococcus aureus*, which recruits host plasma proteins such as fibrinogen and HK to its surface, thereby promoting both bacterial biofilm formation on biomaterial surfaces (Parente et al., 2023), and bradykinin release (Mattsson et al., 2001). Additionally, when *S. epidermidis* was incubated in the presence of DNase I, the aPTT was prolonged and degradation of HK was prevented, supporting an important role of bacterial eDNA in contact activation. Interestingly, DNase I treatment did not markedly reduce PK/FXIIa substrate activity, suggesting that initial PK/FXIIa activation can still occur after cleavage of eDNA, whereas downstream steps of the contact system are affected.

PIA, on the other hand, is a key surface polysaccharide of *S. epidermidis* that mediates intercellular adhesion (Heilmann et al., 1996). In our experiments with planktonic bacteria, the PIA-deficient mutant showed only slightly reduced PK/FXIIa activity; however, similar to the AtlE mutant, HK degradation and aPTT shortening were absent, suggesting that PIA contributes to the downstream steps following FXII and PK activation. Previous work has shown that multiple types of foreign surfaces can trigger FXII and PK activation, although only a subset induce activation of FXI (Maat and Maas, 2016), which is necessary to induce intrinsic coagulation (Schmaier, 2016). FXII contains several surface binding sites, and it was suggested that all of these must be involved in surface binding, as this induces the full conformational change required for FXI binding and activation. When FXII binds only partially to a surface, it can only activate PK (Maat and Maas, 2016).

Based on our results, we propose that eDNA, released via AtlE-mediated lysis, provides a negatively charged scaffold that facilitates partial FXII binding and PK activation, while PIA promotes bacterial aggregates that facilitate full FXII binding and thereby FXI activation. Only when both eDNA and PIA are present, FXI is activated and triggers intrinsic coagulation, leading to aPTT shortening.

Importantly, both eDNA and PIA show significant variability across *S. epidermidis* strains. Clinical isolates differ markedly in their eDNA content (Ravaioli et al., 2011) and biofilm matrix composition, including PIA levels, also ranges widely, even among genetically similar strains (Fernández-Calderón et al., 2025). This phenotypic diversity likely contributes to the strain - specific differences observed in biofilm formation and clotting times in the present study. However, we interpret these findings not as evidence of an evolved bacterial strategy to manipulate host coagulation, but rather as an unintended consequence of *S. epidermidis* presence in an environment, blood plasma, where they are normally absent.

The peptide HKH20, derived from domain 5 of high molecular weight kininogen (Hasan et al., 1995), did not display consistent anti-biofilm activity in terms of biomass reduction. Instead, it did modulate the interaction between *S. epidermidis* and plasma proteins as it effectively inhibited PK/FXIIa activity and prevented degradation of HK. HKH20 also significantly reduced the number and size of bacterial aggregates in plasma and altered biofilm architecture by reducing the formation of a fibrin-rich network. These results suggest that local modulation of plasma protein dynamics, especially fibrin and HK interactions, can influence the spatial structure of bacterial communities, even without directly killing bacteria or inhibiting growth.

Taken together, our data support a model in which coagulation activity, particularly through the contact system, contributes to the architecture and potentially to the stability of *S. epidermidis* biofilms in blood-like environments. This has relevance for medical device-associated infections, where contact between blood, plastic surfaces, and bacteria can lead to parallel activation of coagulation and bacterial adhesion pathways. Strategies that target these host-pathogen interface mechanisms, such as surface coatings that prevent contact activation, may therefore represent promising adjunctive measures for preventing or managing device-related infections.

## Data Availability

The raw data supporting the conclusions of this article will be made available by the authors, without undue reservation.
