# Supplementary material for: Bacterial factors required for biofilm formation in Staphylococcus epidermidis are linked to contact activation
Source: Front Cell Infect Microbiol. 2026 Apr 14;16:1802218. doi: 10.3389/fcimb.2026.1802218 (PMC13121250; doi:10.3389/fcimb.2026.1802218)
Supplement: Supplementary file 1 [file DataSheet1.pdf]

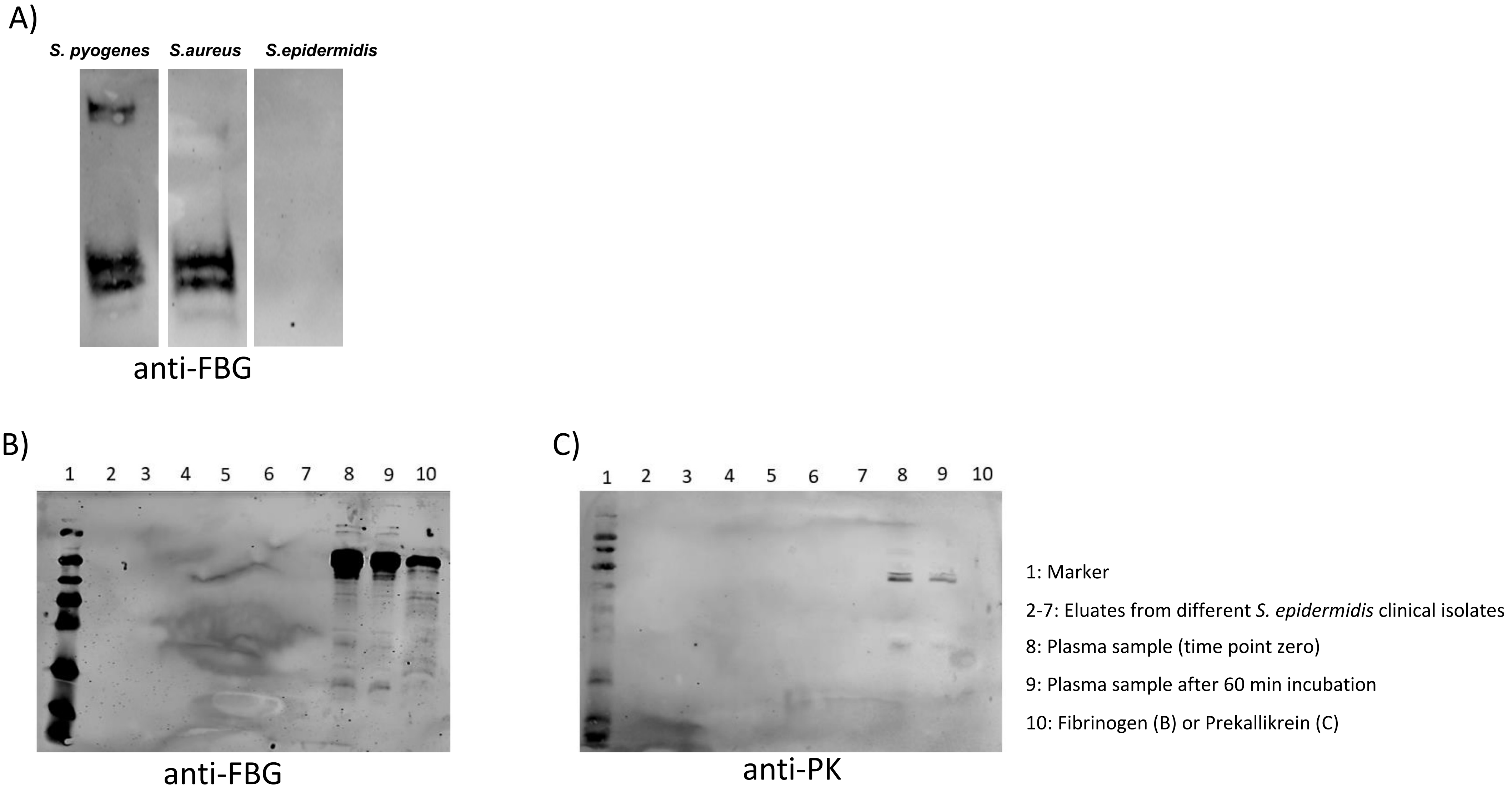

suppl. Figure 1: *S. epidermidis* did not adsorp fibrinogen from plasma. Bacteria were incubated in plasma for 30 min, washed and the adsorpted plasma proteins were eluted from the surface. The eluates were blotted and the membrane was stained with an antibody against fibrinogen (FBG, A and B) or PK (C). A) *S. pyogenes* and *S. aureus* were used as positive controls for fibrinogen aquisition from plasma. B,C) Different clinical S. epidermidis strains were tested negative for adsorption of fibrinogen (B) or plasma kallikrein (C).

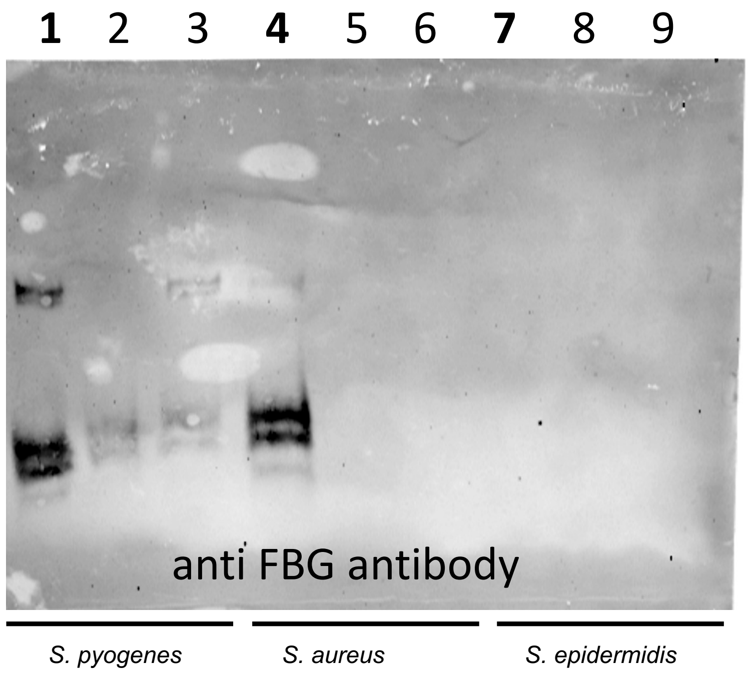

1. **Eluate *Streptococcus pyogenes***
2. Eluate *Streptococcus pyogenes* (different conditions)
3. Eluate *Streptococcus pyogenes* (different conditions)
4. **Eluate *Staphylococcus aureus***
5. Eluate *Staphylococcus aureus* (different conditions)
6. Eluate *Staphylococcus aureus* (different conditions)
7. **Eluate *Staphylococcus epidermidis***
8. Eluate *Staphylococcus epidermidis* (different conditions)
9. Eluate *Staphylococcus epidermidis* (different conditions)

Suppl. Figure 2: Full uncropped blot corresponding to Supplementary Figure S1A. The regions displayed in Supplementary Figure S1 are indicated in bold. Additional lanes shown in the full blot correspond to samples or experimental conditions not included in the final analysis.
